# Supplementary material for: Methods for the inclusion of real-world evidence in network meta-analysis
Source: BMC Med Res Methodol. 2021 Oct 9;21:207. doi: 10.1186/s12874-021-01399-3 (PMC8502389; doi:10.1186/s12874-021-01399-3)
Supplement: Supplementary file 1 — Additional file 1. Search terms used for the systematic review assessing the impact of treatments in relapsing remitting multiple sclerosis. [file 12874_2021_1399_MOESM1_ESM.docx]

# Additional File 1

**Search terms used for the systematic review assessing the impact of treatments in relapsing remitting multiple sclerosis**

| S**tep** | **Terms Used** |
| --- | --- |
| 1 | Multiple Sclerosis, Relapsing-Remitting/ or multiple sclerosis.sh,tw. |
| 2 | (relapsing remitting adj2 multiple sclerosis).ti,ab,sh,hw,ot. |
| 3 | (remitting relapsing adj2 multiple sclerosis).ti,ab,sh,hw,ot. |
| 4 | ((relapsing remitting adj2 ms) or (remitting relapsing adj2 ms)).ti,ab,sh,hw,ot. |
| 5 | ((exacerbat* or disseminated or insular or secondary progressive or primary progressive or progressive relapsing) adj2 (sclerosis or ms)).ti,ab,sh,hw,ot. |
| 6 | (rrms or encephalomyelitis disseminat*).ti,ab,sh,hw,ot. |
| 7 | or/1-6 |
| 8 | (teriflunomide or A 1726 or A 77 1726 or A 771726 or HMR 1726 or HMR1726 or aubagio or 163451-81-8).ti,ab,rn,nm,sh,hw,ot. |
| 9 | (fingolimod or FTY-720 or FTY270 or Gilenya or Gilenia or 162359-55-9).ti,ab,rn,nm,sh,hw,ot. |
| 10 | (natalizumab or tysabri or antegren or 189261-10-7).ti,ab,rn,nm,sh,hw,ot. |
| 11 | or/8-10 |
| 12 | Interferon-beta/ |
| 13 | (interferon-beta-1 or interferon-1a or interferon-1b or interferon beta or beta Interferon or Interferon beta 1 or 220581-49-7 or 145155-23-3).ti,ab,rn,nm,sh,hw,ot. |
| 14 | (avonex or rebif or betaferon or betaseron or BAY 86-5046 or BAY86-5046 or extavia).ti,ab,rn,nm,sh,hw,ot. |
| 15 | (glatiramer acetate or copaxone or 147245-92-9).ti,ab,rn,nm,sh,hw,ot. |
| 16 | (fampridine or fampyra or 504-24-5).ti,ab,rn,nm,sh,hw,ot. |
| 17 | or/12-16 |
| 18 | exp epidemiologic studies/ or (epidemiolog* adj (study or studies)).tw. |
| 19 | Observational Study.pt. or ((observational or cohort or case-control or case control or cross sectional or cross-sectional) adj (study or studies)).tw. |
| 20 | ((follow up or follow-up) adj2 (study or studies)).tw. |
| 21 | (longitudinal or retrospective or prospective).tw. |
| 22 | or/18-21 |
| 23 | Comparative study.pt. or (comparative adj (study or studies)).tw. |
| 24 | Multicenter Study.pt. |
| 25 | Pragmatic Clinical Trial.pt. or (pragmatic$ adj3 (study or studies or trial$)).tw. |
| 26 | Clinical Trial, Phase IV.pt. or (phase adj ((relapsing remitting adj2 ms) or (remitting relapsing adj2 ms) or IV or four) adj (study or studies or trial$)).tw. |
| 27 | exp Questionnaires/ |
| 28 | Health Care Surveys/ |
| 29 | Health Surveys/ |
| 30 | Registries/ or registr*.tw. |
| 31 | Records as Topic/ or (((hospital or medical) adj records) or (chart$ adj3 review$)).tw. |
| 32 | (database adj5 (study or studies or report* or research or activit*)).tw. |
| 33 | ((monitoring or surveillance or extension) adj3 (data or study or studies or report* or trial*)).tw. |
| 34 | or/23-33 |
| 35 | Meta-Analysis.pt. |
| 36 | meta-analysis/ or systematic review/ or meta-analysis as topic/ or "meta analysis (topic)"/ or "systematic review (topic)"/ or exp technology assessment, biomedical/ |
| 37 | (met analy* or metanaly* or technology assessment* or HTA or HTAs or technology overview*).ti,ab. |
| 38 | (meta-analy* or metaanaly* or systematic review* or biomedical technology assessment* or bio-medical technology assessment*).mp,hw. |
| 39 | or/35-38 |
| 40 | ((real world adj (data or evidence)) or relative effectiveness).tw. |
| 41* | 7 and (22 or 34 or 39 or 40) and 8 - TERIFLUNOMIDE |
| 42* | 7 and (22 or 34 or 39 or 40) and 9 - FINGOLIMOD |
| 43** | 7 and (22 or 34 or 39 or 40) and 10 - NATALIZUMAB |
| 44* | 7 and (22 or 34 or 39 or 40) and 12 and 13 and 14 - INTERFERON BETAS |
| 45* | 7 and (22 or 34 or 39 or 40) and 15 - GLATIRAMER ACETATE |
| 46* | 7 and (22 or 34 or 39 or 40) and 16 - FAMPRIDINE |
