# Supplementary material for: Methods for the inclusion of real-world evidence in network meta-analysis
Source: BMC Med Res Methodol. 2021 Oct 9;21:207. doi: 10.1186/s12874-021-01399-3 (PMC8502389; doi:10.1186/s12874-021-01399-3)
Supplement: Supplementary file 2 — Additional file 2. Reference list of randomised controlled trials and real-world studies include in the network meta-analysis assessing the impact of treatments in relapsing remitting multiple sclerosis. [file 12874_2021_1399_MOESM2_ESM.docx]

# Additional File 2

**Reference list of randomised controlled trials and real-world studies include in the network meta-analysis assessing the impact of treatments in relapsing remitting multiple sclerosis**

1. Polman, C., O’Connor, P., Havrdova, E., Hutchinson, M., Kappos, L., Miller, D., Phillips, J., Lublin, F., Giovannoni, G., Wajgt, A., Toal, M., Lynn, F., Panzara, M. and Sandrock, A. (2006), ‘A randomized, placebo-controlled trial of natalizumab for relapsing multiple sclerosis’, *The New England Journal of Medicine* **354**, 899–910.
2. Cohen, J., Barkhof, F., Comi, G., Hartung, H., Khatri, B., Montalban, X., Pelletier, J., Capra, R., Gallo, P., Izquierdo, G., Tiel-Wilck, K., de Vera, A., Jin, J., Stites, T., Wu, S., Aradhye, S. and Kappos, L. (2010), ‘Oral fingolimod or intramuscular interferon for relapsing multiple sclerosis’, *The New England Journal of Medicine* **362**(362), 402–415.
3. Johnson, K., Brooks, B., Cohen, J., Ford, C., Goldstein, J., Lisak, R., Myers, L., Panitch, H., Rose, J., Schiffer, R., Vollmer, T., Weiner, L. and Wolinsky, J. (1995), Copolymer 1 reduces relapse rate and improves disability in relapsing-remitting multiple sclerosis:Results of a phase 3 multicenter, double-blind, placebo-controlled trial’, *Neurology* **45**(7), 1268–1276.
4. Limmroth, V., Malessa, R., Zettl, U.K., Koehler, J., Japp, G., Haller, P., Elias, W., Obhof, W., Viehöver, A. & Meier, U. 2007, "Quality assessment in multiple sclerosis therapy (Quasims)", *Journal of neurology,* vol. 254, no. 1, pp. 67-77.
5. Halpern, R., Agarwal, S., Borton, L., Oneacre, K. & Lopez-Bresnahan, M.V. 2011, "Adherence and persistence among multiple sclerosis patients after one immunomodulatory therapy failure: retrospective claims analysis", *Advances in Therapy,* vol. 28, no. 9, pp. 761-775.
6. Haas, J. and Firzlaff, M. (2005), ‘Twenty-four-month comparison of immunomodulatory treatments a retrospective open label study in 308 RRMS patients treated with beta interferons or glatiramer acetate (copaxone)’, *European Journal of Neurology* **12**(6), 425–431.
7. IFNB Group (1993), ‘Interferon beta-1b is effective in relapsing-remitting multiple sclerosis: I. clinical results of a multicenter, randomized, double-blind, placebo-controlled trial’, *Neurology* **43**, 655–661.
8. Jacobs, L., Cookfair, D., Rudick, R., Herndon, R., Richert, J., Salazar, A., Fischer, J.,Goodkin, D., Granger, C., Simon, J., Alam, J., Bartoszak, D., Bourdette, D., Braiman, J., Brownscheidle, C., Coats, M., Cohan, S., Dougherty, D., Kinkel, R., Mass, M., Munschauer, F., Priore, R., Pullicino, P., Scherokman, B., Weinstock-Guttman, B., Whitham, R. and The Multiple Sclerosis Collaborative Research Group (MSCRG) (1996), ‘Intramuscular interferon beta-1a for disease progression in relapsing multiple sclerosis’, *Annals of Neurology* **39**(3), 285–294.
9. Khan, O., Tselis, C., Kamholz, J., Garbern, J., Lewis, R. and Lisak, R. (2001), ‘A prospective, open-label treatment trial to compare the effect of IFNb-1a (Avonex), IFNb-1b (Betaseron), and glatiramer acetate (Copaxone) on the relapse rate in relapsing-remitting multiple sclerosis: results after 18 months of therapy’, *Multiple Sclerosis* **7**(6), 349–353.
10. Kappos, L., Radue, E., O’Connor, P., Polman, C., Hohlfeld, R., Calabresi, P., Selmaj, K., Agoropoulou, C., Leyk, M., Zhang-Auberson, L. and Burtin, P. (2010), ‘A placebocontrolled trial of oral fingolimod in relapsing multiple sclerosis’, *The New England Journal of Medicine* **362**, 387–401.
11. O’Connor, P., Filippi, M., Arnason, B., Comi, G., Cook, S., Goodin, D., Hartung, H., Jefferey, D., Kappos, L., Boateng, F., Fillipov, V., Groth, M., Knappertz, V., Kraus, C., Sandbrink, R., Pohl, C. and Bogumil, T. (2009), ‘250 *_*g or 500 *_*g interferon beta-1b versus 20 mg glatiramer acetate in relapsing-remitting multiple sclerosis: a prospective, randomised, multicentre study’, *The Lancet, Neurology* **8**(10), 889–897.
12. PRISMS Group (1998), ‘Randomised double-blind placebo-controlled study of interferon beta-1a in relapsing/remitting multiple sclerosis’, *The Lancet* **352**, 1498–1504.
13. Panitch, H., Goodin, D., Francis, G., Chang, P., Coyle, P., OConnor, P., Monaghan, E., Li, D. and Weinshenker, B. (2002), ‘Randomized, comparative study of interferon *beta*-1a treatment regimens in MS: The EVIDENCE trial’, *Neurology* **59**(10), 1496–1506.
14. Mikol, D., Barkhof, F., Chang, P., Coyle, P., Jeffery, D., Schwid, S., Stubinski, B. and Uitdehaag, B. (2008), ‘Comparison of subcutaneous interferon beta-1a with glatiramer acetate in patients with relapsing multiple sclerosis (the rebif vs glatiramer acetate in relapsing ms disease [REGARD] study): a multicentre, randomised, parallel, openlabel trial’, *The Lancet Neurology* **7**(10), 903–914.
15. Trojano, M., Liguori, M., Paolicelli, M., Bosco Zimatore, G., De Robertis, F., Avolio, C., Giuliani, F., Fuiani, A. and Livrea, P. (2003), ‘Interferon beta in relapsing/remitting multiple sclerosis: an independent postmarketing study in southern Italy’, *Multiple* *Sclerosis* **9**(5), 451–457.
16. Comi, G., Filippi, M., Wolinsky, J. and European/Canadian Glatiramer Acetate Study Group (2001), ‘European/Canadian multicenter, double-blind, randomized, placebocontrolled study of the effects of glatiramer acetate on magnetic resonance imagingmeasured disease activity and burden in patients with relapsing multiple sclerosis’, *Annals of Neurology* **49**(3), 290–297.
17. Durelli, L., Verdun, E., Barbero, P., Bergui, M., Versino, E., Ghezzi, A., Montanari, E. and Zaffaroni, M. (2002), ‘Every-other-day interferon beta-1b versus once-weekly interferon beta-1a for multiple sclerosis: results of a 2-year prospective randomised multicentre study (INCOMIN)’, *The Lancet* **359**(9316), 1453–1460.
18. Carra, A., Onaha, P., Sinay, V., Alvarez, F., Luetic, G., Bettinelli, R., San Pedro, E. and Rodriguez, L. (2003), ‘A retrospective, observational study comparing the four available immunomodulatory treatments for relapsing-remitting multiple sclerosis’, *European* *Journal of Neurology* **10**(6), 671–676.
19. Cadavid, D., Wolansky, L., Skurnick, J., Lincoln, J., Cheriyan, J., Szczepanowski, K., Kamin, S., Pachner, A., Halper, J. and Cook, S. (2009), ‘Efficacy of treatment of MS with IFN*_*-1b or glatiramer acetate by monthly brain MRI in the BECOME study’, *Neurology* **72**(23), 1976–1983.
20. Calabresi, P. A., Radue, E. W., Goodin, D., Jeffery, D., Rammohan, K. W., Reder, A. T., ... & Lublin, F. D. (2014). Safety and efficacy of fingolimod in patients with relapsing-remitting multiple sclerosis (FREEDOMS II): a double-blind, randomised, placebo-controlled, phase 3 trial. *The Lancet Neurology*, *13*(6), 545-556.
21. Río, Jordi, et al. "Evaluating the response to glatiramer acetate in relapsing–remitting multiple sclerosis (RRMS) patients." Multiple Sclerosis Journal (2014): 1352458514527863.
22. Lanzillo, R., et al. "Natalizumab vs interferon beta 1a in relapsing‐remitting multiple sclerosis: a head‐to‐head retrospective study." *Acta Neurologica Scandinavica* 126.5 (2012): 306-314.
23. Patti, F., Pappalardo, A., Florio, C., Politi, G., Fiorilla, T., Reggio, E. & Reggio, A. 2006, "Effects of interferon beta-1a and -1b over time: 6-year results of an observational head-to-head study", *Acta Neurologica Scandinavica,* vol. 113, no. 4, pp. 241-247.
