# Supplementary material for: Methods for the inclusion of real-world evidence in network meta-analysis
Source: BMC Med Res Methodol. 2021 Oct 9;21:207. doi: 10.1186/s12874-021-01399-3 (PMC8502389; doi:10.1186/s12874-021-01399-3)
Supplement: Supplementary file 3 — Additional file 3. Number of subjects, number of relapses and exposure time (person-years) extracted and analysed from randomised controlled trials and real-world studies assessing the impact of treatments in relapse remitting multiple sclerosis. [file 12874_2021_1399_MOESM3_ESM.docx]

# Additional File 3

**Number of subjects, number of relapses and exposure time (person-years) extracted and analysed from randomised controlled trials and real-world studies assessing the impact of treatments in relapse remitting multiple sclerosis**

| **First author** | **Treatment** | **No of subjects** | **No of relapses** | **Exposure time (person-years)** |
| --- | --- | --- | --- | --- |
| **Randomised controlled trials** | | | | |
| **Polman et al. (2006)** | Placebo | 315 | 433 | 593 |
|  | Natalizumab | 627 | 276 | 1202 |
| **Kappos et al. (2010)** | Placebo | 418 | 300 | 750 |
|  | Fingolimod (1.25mg) | 429 | 122 | 762 |
|  | Fingolimod (0.5mg) | 425 | 143 | 794 |
| **Cohen et al. (2010)** | Avonex | 431 | 135 | 409 |
|  | Fingolimod (1.25mg) | 420 | 79 | 395 |
|  | Fingolimod (0.5mg) | 429 | 66 | 414 |
| **Freedoms (2010)** | Placebo | 355 | 0 | 710 |
|  | Fingolimod (0.5mg) | 358 | 150 | 716 |
|  | Fingolimod (1.25mg) | 370 | 0 | 740 |
| **PRISMS Group (1998)** | Placebo | 187 | 479 | 364 |
|  | Rebif 22 | 189 | 344 | 366 |
|  | Rebif 44 | 184 | 318 | 363 |
| **Johnson et al. (1995)** | Placebo | 126 | 210 | 250 |
|  | Copaxone | 125 | 161 | 273 |
| **Comi et al. (2001)** | Placebo | 120 | 91 | 75 |
|  | Copaxone | 119 | 61 | 75 |
| **Jacobs et al. (1996)** | Placebo | 143 | 225 | 274 |
|  | Avonex | 158 | 196 | 293 |
| **Durelli et al. (2002)** | Avonex | 92 | 126 | 180 |
|  | Betaferon | 96 | 95 | 190 |
| **Panitch et al. (2002)** | Avonex | 338 | 216 | 304 |
|  | Rebif 44 | 339 | 183 | 305 |
| **IFNB Group (1993)** | Placebo | 123 | 266 | 209 |
|  | Betaferon | 124 | 173 | 206 |
| **O’Connor et al. (2009)** | Copaxone | 448 | 383 | 1126 |
|  | Betaferon | 897 | 828 | 2299 |
| **O’Connor et al. (2009)** | Copaxone | 39 | 23 | 70 |
|  | Betaferon | 36 | 25 | 68 |
| **Mikol et al. (2008)** | Copaxone | 386 | 200 | 688 |
|  | Rebif 44 | 387 | 207 | 689 |
| **Real-world evidence** | | | | |
| **Lanzillo (2010/2011)** | Natalizumab | 42 | 10 | 42 |
|  | Rebif 44 | 42 | 23 | 42 |
| **Limmroth (2007)** | Avonex | 1094 | 1116 | 2188 |
| **(QUASIMS)** | Betaferon | 1034 | 1075 | 2068 |
|  | Rebif 22 | 555 | 588 | 1110 |
|  | Rebif 44 | 185 | 233 | 370 |
| **Halpern 2011** | Natalizumab | 288 | 21 | 72 |
|  | Avonex | 151 | 7 | 38 |
|  | Rebif 22 | 329 | 22 | 82 |
|  | Betaferon | 144 | 11 | 36 |
|  | Copaxone | 469 | 25 | 117 |
| **Patti (2006)** | Betaferon | 114 | 137 | 570 |
|  | Avonex | 37 | 50 | 185 |
|  | Rebif 22 | 17 | 35 | 85 |
| **Rio (2005)** | Placebo | 107 | 288 | 356 |
|  | Copaxone | 101 | 204 | 334 |
| **Haas and Firzlaff (2005)** | Avonex | 79 | 109 | 158 |
|  | Betaferon | 77 | 123 | 154 |
|  | Copaxone | 79 | 56 | 158 |
|  | Rebif 22 | 48 | 59 | 96 |
| **Khan et al. (2001)** | Placebo | 15 | 23 | 23 |
|  | Avonex | 34 | 41 | 51 |
|  | Betaferon | 34 | 28 | 51 |
|  | Copaxone | 39 | 29 | 59 |
| **Trojano et al. (2003)** | Betaferon | 209 | 136 | 418 |
|  | Avonex | 169 | 120 | 338 |
| **Carra et al. (2003)** | Avonex | 26 | 14 | 35 |
|  | Rebif 44 | 20 | 12 | 27 |
|  | Betaferon | 20 | 11 | 27 |
|  | Copaxone | 30 | 8 | 40 |
