# Supplementary material for: Methods for the inclusion of real-world evidence in network meta-analysis
Source: BMC Med Res Methodol. 2021 Oct 9;21:207. doi: 10.1186/s12874-021-01399-3 (PMC8502389; doi:10.1186/s12874-021-01399-3)
Supplement: Supplementary file 4 — Additional file 4. Annualised relapse rate ratios (95% credible intervals) of each active treatment compared to placebo for values alpha using the power prior model with between study heterogeneity standard deviation estimates. [file 12874_2021_1399_MOESM4_ESM.docx]

# Additional File 4

**Annualised relapse rate ratios (95% credible intervals) of each active treatment compared to placebo for values alpha using the power prior model with between study heterogeneity standard deviation estimates**

|  | | | | | | | | | | |
| --- | --- | --- | --- | --- | --- | --- | --- | --- | --- | --- |
| Alpha | **Natalizumab** | **Fingolimod 1.25mg** | **Fingolimod 0.5mg** | **Avonex** | **Rebif 22** | **Rebif 44** | **Copaxone** | **Betaferon** | **Between study SD** |  |
| 0.001 | 0.32 (0.26, 0.39) | 0.46 (0.40, 0.54) | 0.42 (0.36, 0.50) | 0.83 (0.72, 0.95) | 0.71 (0.60, 0.86) | 0.68 (0.59, 0.78) | 0.65 (0.57, 0.75) | 0.67 (0.58, 0.77) | 0.055 |  |
| 0.1 | 0.32 (0.27, 0.39) | 0.46 (0.40, 0.53) | 0.42 (0.36, 0.48) | 0.79 (0.71, 0.90) | 0.73 (0.64, 0.85) | 0.69 (0.61, 0.78) | 0.66 (0.58, 0.75) | 0.68 (0.60, 0.78) | 0.045 |  |
| 0.2 | 0.33 (0.27, 0.41) | 0.46 (0.39, 0.53) | 0.42 (0.36, 0.50) | 0.78 (0.70, 0.88) | 0.72 (0.62, 0.84) | 0.69 (0.61, 0.79) | 0.65 (0.57, 0.73) | 0.68 (0.61, 0.77) | 0.047 |  |
| 0.3 | 0.33 (0.27, 0.42) | 0.45 (0.39, 0.54) | 0.42 (0.35, 0.49) | 0.77 (0.67, 0.88) | 0.73 (0.64, 0.84) | 0.70 (0.62, 0.81) | 0.65 (0.58, 0.74) | 0.69 (0.60, 0.78) | 0.057 |  |
| 0.4 | 0.34 (0.27, 0.43) | 0.45 (0.38, 0.54) | 0.41 (0.35, 0.50) | 0.77 (0.68, 0.89) | 0.74 (0.63, 0.87) | 0.72 (0.62, 0.84) | 0.65 (0.56, 0.74) | 0.69 (0.61, 0.80) | 0.085 |  |
| 0.5 | 0.35 (0.28, 0.46) | 0.46 (0.38, 0.55) | 0.42 (0.34, 0.50) | 0.78 (0.68, 0.90) | 0.75 (0.63, 0.89) | 0.72 (0.62, 0.85) | 0.64 (0.55, 0.73) | 0.70 (0.61, 0.81) | 0.100 |  |
| 0.6 | 0.37 (0.29, 0.50) | 0.46 (0.37, 0.57) | 0.41 (0.33, 0.51) | 0.78 (0.67, 0.92) | 0.75 (0.62, 0.91) | 0.73 (0.61, 0.88) | 0.63 (0.53, 0.73) | 0.70 (0.59, 0.82) | 0.131 |  |
| 0.7 | 0.38 (0.29, 0.53) | 0.46 (0.36, 0.57) | 0.42 (0.33, 0.52) | 0.78 (0.67, 0.92) | 0.75 (0.62, 0.93) | 0.73 (0.61, 0.89) | 0.62 (0.52, 0.72) | 0.70 (0.59, 0.82) | 0.144 |  |
| 0.8 | 0.39 (0.29, 0.54) | 0.46 (0.36, 0.58) | 0.41 (0.32, 0.53) | 0.78 (0.66, 0.93) | 0.76 (0.62, 0.94) | 0.74 (0.61, 0.91) | 0.61 (0.51, 0.72) | 0.70 (0.58, 0.83) | 0.162 |  |
| 0.9 | 0.40 (0.30, 0.56) | 0.46 (0.35, 0.59) | 0.41 (0.32, 0.53) | 0.78 (0.65, 0.94) | 0.76 (0.61, 0.95) | 0.74 (0.60, 0.92) | 0.61 (0.51, 0.72) | 0.70 (0.58, 0.83) | 0.173 |  |
| 1.0 | 0.41 (0.30, 0.57) | 0.45 (0.35, 0.59) | 0.41 (0.32, 0.53) | 0.78 (0.65, 0.93) | 0.76 (0.61, 0.95) | 0.74 (0.60, 0.93) | 0.60 (0.49, 0.71) | 0.69 (0.57, 0.83) | 0.182 |  |
