# Supplementary material for: Methods for the inclusion of real-world evidence in network meta-analysis
Source: BMC Med Res Methodol. 2021 Oct 9;21:207. doi: 10.1186/s12874-021-01399-3 (PMC8502389; doi:10.1186/s12874-021-01399-3)
Supplement: Supplementary file 5 — Additional file 5. Annualised relapse rate ratios (95% credible intervals) of each active treatment compared to placebo for values of the down-weighting factor (alpha) between zero (total down-weighting, i.e. RWE not included) and one (RWE considered at ‘face-value’) using the hierarchical power prior model. [file 12874_2021_1399_MOESM5_ESM.docx]

# Additional File 5

**Annualised relapse rate ratios (95% credible intervals) of each active treatment compared to placebo for values of the down-weighting factor (alpha) between zero (total down-weighting, i.e. RWE not included) and one (RWE considered at ‘face-value’) using the hierarchical power prior model**

| **Alpha** | **Natalizumab** | **Fingolimod 1.25mg** | **Fingolimod 0.5mg** | **Avonex** | **Rebif 22** | **Rebif 44** | **Copaxone** | **Betaferon** |
| --- | --- | --- | --- | --- | --- | --- | --- | --- |
| **0.001** | 0.35 (0.14, 0.74) | 0.46 (0.40, 0.54) | 0.42 (0.36, 0.49) | 0.88 (0.44, 1.60) | 0.79 (0.39, 1.56) | 0.76 (0.40, 1.50) | 0.68 (0.34, 1.22) | 0.74 (0.39, 1.41) |
| **0.1** | 0.33 (0.24, 0.50) | 0.46 (0.40, 0.53) | 0.42 (0.36, 0.49) | 0.81 (0.61, 1.07) | 0.75 (0.57, 1.04) | 0.73 (0.56, 1.05) | 0.65 (0.48, 0.84) | 0.70 (0.54, 0.97) |
| **0.2** | 0.35 (0.25, 0.54) | 0.46 (0.40, 0.53) | 0.42 (0.36, 0.49) | 0.80 (0.60, 1.09) | 0.75 (0.55, 1.02) | 0.74 (0.56, 1.07) | 0.63 (0.46, 0.84) | 0.71 (0.53, 0.98) |
| **0.3** | 0.35 (0.25, 0.56) | 0.46 (0.40, 0.53) | 0.42 (0.36, 0.49) | 0.81 (0.60, 1.10) | 0.75 (0.56, 1.04) | 0.74 (0.56, 1.07) | 0.63 (0.46, 0.83) | 0.71 (0.53, 0.97) |
| **0.4** | 0.37 (0.25, 0.61) | 0.46 (0.40, 0.54) | 0.42 (0.36, 0.49) | 0.82 (0.59, 1.18) | 0.77 (0.55, 1.12) | 0.77 (0.56, 1.17) | 0.63 (0.44, 0.87) | 0.72 (0.52, 1.05) |
| **0.5** | 0.37 (0.26, 0.59) | 0.46 (0.40, 0.54) | 0.42 (0.36, 0.49) | 0.82 (0.59, 1.17) | 0.77 (0.55, 1.11) | 0.77 (0.56, 1.17) | 0.63 (0.44, 0.87) | 0.72 (0.52, 1.03) |
| **0.6** | 0.40 (0.26, 0.73) | 0.46 (0.40, 0.54) | 0.42 (0.36, 0.49) | 0.85 (0.55, 1.40) | 0.80 (0.52, 1.32) | 0.81 (0.54, 1.37) | 0.64 (0.41, 1.00) | 0.75 (0.49, 1.24) |
| **0.7** | 0.40 (0.26, 0.73) | 0.46 (0.40, 0.54) | 0.42 (0.36, 0.49) | 0.84 (0.55, 1.36) | 0.79 (0.52, 1.29) | 0.81 (0.54, 1.37) | 0.63 (0.41, 0.98) | 0.74 (0.49, 1.22) |
| **0.8** | 0.39 (0.26, 0.68) | 0.46 (0.39, 0.54) | 0.42 (0.36, 0.49) | 0.83 (0.55, 1.26) | 0.77 (0.52, 1.19) | 0.78 (0.53, 1.26) | 0.62 (0.41, 0.91) | 0.72 (0.49, 1.11) |
| **0.9** | 0.40 (0.26, 0.68) | 0.46 (0.40, 0.54) | 0.42 (0.36, 0.49) | 0.82 (0.55, 1.25) | 0.77 (0.51, 1.21) | 0.79 (0.53, 1.27) | 0.62 (0.40, 0.92) | 0.72 (0.49, 1.11) |
| **1.0** | 0.40 (0.26, 0.70) | 0.46 (0.40, 0.54) | 0.42 (0.36, 0.49) | 0.83 (0.55, 1.26) | 0.78 (0.51, 1.21) | 0.79 (0.53, 1.27) | 0.62 (0.40, 0.92) | 0.72 (0.48, 1.13) |
